# Evaluation of Plasma Opioid and Serum Lipocalin-2 Levels in Pruritic Skin Diseases

**DOI:** 10.14789/ejmj.JMJ24-0043-OA

**Published:** 2025-04-18

**Authors:** MAYU KAWAMURA, YAYOI KAMATA, ERIKO KOMIYA, CATHARINA SAGITA MONIAGA, NOBUAKI TAKAHASHI, TAKAHIDE KANEKO, YASUSHI SUGA, MITSUTOSHI TOMINAGA, KENJI TAKAMORI

**Affiliations:** 1 Juntendo Itch Research Center (JIRC), Institute for Environment and Gender-Specific Medicine, Juntendo University Graduate School of Medicine, Chiba, Japan; 1 Juntendo Itch Research Center (JIRC), Institute for Environment and Gender-Specific Medicine, Juntendo University Graduate School of Medicine, Chiba, Japan; 2 Department of Dermatology, Juntendo University Urayasu Hospital, Chiba, Japan; 2 Department of Dermatology, Juntendo University Urayasu Hospital, Chiba, Japan; 3 Anti-Aging Skin Research Laboratory, Juntendo University Graduate School of Medicine, Chiba, Japan; 3 Anti-Aging Skin Research Laboratory, Juntendo University Graduate School of Medicine, Chiba, Japan

**Keywords:** dynorphin A, β-endorphin, lipocalin-2, pruritic skin diseases, VAS score

## Abstract

**Objectives:**

Although the opioid system is critical for regulating itch within the central nervous system, its role in managing severe, treatment-resistant itch related to skin diseases is unclear. Serum lipocalin-2 (LCN-2) levels are associated with the visual analogue scale score of itch in patients with psoriasis vulgaris (PSO), but not other itchy skin diseases. Therefore, we herein investigated the relationship between opioids and LCN-2 with itch in pruritic skin diseases.

**Methods:**

We assessed plasma levels of β-endorphin and dynorphin A and serum levels of LCN-2 in patients with pruritic skin diseases using enzyme-linked immunosorbent assays.

**Results:**

Plasma dynorphin A levels were significantly reduced in patients with urticaria, and asteatotic dermatitis (AsD), while β-endorphin levels were significantly elevated in those with AsD. The plasma β-endorphin-to-dynorphin A ratio was markedly higher in AsD patients than in healthy controls. Among the patients examined, only those with PSO had significantly elevated serum LCN-2 levels, which correlated with VAS and severity index scores and also with the area of affected skin.

**Conclusions:**

The present results suggest that the ratio of plasma opioids is involved in itch control in AsD patients, and serum LCN-2 levels may be a biomarker for itch and its severity in those with PSO.

## Introduction

Pruritus, a frequently recurring symptom in dermatologic conditions, diminishes patients' quality of life. It is generally categorized into two main types: peripheral and central, with central pruritus being linked to opioid system involvement^1)^. Three primary opioid receptor types have been identified: μ-type (MOR, β-endorphin receptor), κ-type (KOR, dynorphin receptor), and δ-type (enkephalin receptor)^2)^. μ- and κ-opioid receptors are key players in the regulation of itch within the central nervous system (CNS). The activation of μ-opioid receptors is considered to trigger itch, while the activation of κ-opioid receptors appears to suppress it^3, 4)^. Opioid- induced pruritus is a well-documented side effect associated with morphine and other μ-opioid receptor agonists used in pain management^5, 6)^. Previous studies suggested that the κ-opioid system helped reduce persistent itch in patients with kidney and chronic liver diseases^7, 8)^.

Among patients skin diseases, elevated serum β-endorphin levels have been observed in those with widespread lesions, including atopic dermatitis (AD), systemic scleroderma, and particularly psoriasis vulgaris (PSO)^9)^. Additionally, the treatment of senile pruritus with the Guishen Zhiyang Recipe markedly reduced serum dynorphin levels^10)^. However, the involvement of opioid systems in the regulation of persistent, treatment-resistant itch in skin disorders remains unclear.

Lipocalin-2 (LCN-2), also referred to as 24p3 and neutrophil gelatinase-binding lipocalin, is primarily secreted by activated neutrophils^11)^. LCN-2 is synthesized as a glycosylated 25-kDa monomer, but is typically found in the bloodstream as a dimeric or larger multimer, often forming a stable complex with the protease matrix metalloproteinase-9 (MMP- 9)^11)^. Serum LCN-2 levels have been reported to correlate with visual analogue scale (VAS) scores for itch in patients with PSO^12)^, but not other pruritic skin conditions.

In the present study, we measured plasma levels of dynorphin A and β-endorphin as well as serum levels of LCN-2, MMP-9, and the MMP-9/LCN-2 complex and investigated their relationships with itch severity in pruritic skin diseases, such as AD, PSO, prurigo nodularis (PN), urticaria, palmoplantar pustulosis (PPP), and asteatotic dermatitis (AsD). Serum levels were assessed using enzyme-linked immunosorbent assays (ELISA) and compared with VAS scores for itch.

## Materials and Methods

### Human samples

Peripheral blood samples were collected from patients with pruritic skin conditions, including AD, PSO, PN, urticaria, PPP, and AsD, and those receiving hemodialysis. Itch severity was assessed using VAS. After collection, blood samples were centrifuged at 3,000 rpm at 4℃ for 20 min, and plasma was stored at -80℃. None of the patients had received oral μ-opioid antagonists (e.g., naloxone and naltrexone), κ-opioid receptor agonists (e.g., nalfurafine hydrochloride), or opioid pain medications prior to sampling. The Juntendo University Urayasu Hospital Ethics Committee approved the study protocol (U13-0003-U01). Written informed consent was obtained from all patients and the study complied with the Declaration of Helsinki.

Patients assessed for dynorphin A and β-endorphin levels (n = 110; sex = 60 males (m), 50 females (f); age: mean ± SD, 52.4 ± 20.3, range 17-92 yrs) were categorized into seven groups: 21 healthy controls (33.3 ± 8.3 yrs), 19 AD patients (38.4 ± 13 yrs), 10 with PSO (61.5 ± 11.9 yrs), 13 with PN (55 ± 14.9 yrs), 15 with urticaria (50.7 ± 20.3 yrs), 11 with PPP (60.7 ± 16.3 yrs), 10 with AsD (79.5 ± 9.5 yrs), and 11 receiving hemodialysis (70.6 ± 14.8 yrs) (Table 1).

Patients assessed for LCN-2, MMP-9, and MMP- 9/ LCN-2 complex levels (n = 76; sex = 43 m, 33 f; age: mean ± SD, 49.7 ± 17.8, range 17-86 yrs) were categorized into six groups: eight healthy controls (35.4 ± 11.4 yrs), 19 AD patients (38.4 ± 13 yrs), 10 with PSO (61.5 ± 11.9 yrs), 13 with PN (55 ± 14.9 yrs), 15 with urticaria (50.7 ± 20.3 yrs), and 11 with PPP (60.7 ± 16.3 yrs) (Table 1).

Sample volumes were partially insufficient for LCN-2, MMP-9, and MMP-9/LCN-2 analyses; therefore, the number of healthy controls decreased from 21 to 8, and it was not possible to analyze their levels in patients with AsD or those receiving hemodialysis.

**Table:** Demographic characteristics of subjects

	HC （Opioid analysis）	HC (LCN-2 analysis)	AD	PSO	PN	Urticaria	PPP	AsD (Opioid analysis)	Hemodialysis (Opioid analysis)
Age									
Mean	33.3 ± 8.3	35.4 ± 11.4	38.4 ± 13	61.5 ± 11.9	55 ± 14.9	50.7 ± 20.3	60.7 ± 16.3	79.5 ± 9.5	70.6 ± 14.8
Range	26 to 43	28 to 59	17 to 62	39 to 80	24 to 75	22 to 86	19 to 81	57 to 92	29 to 88
Sex									
Male	3	2	18	8	6	5	4	9	7
Female	18	6	1	2	7	10	7	1	4
Total number	21	8	19	10	13	15	11	10	11
PASI									
≤ 10				5 (2.4 to 8.3)					
> 10				5 (10.2 to 38.4)					

*Healthy controls (HC); Atopic dermatitis (AD); Psoriasis vulgaris (PSO); Prurigo nodularis (PN); Palmoplantar pustulosis (PPP); Asteatotic dermatitis (AsD) **Psoriasis Area Severity Index (PASI)

### Extraction of peptides from plasma

To quantify dynorphin A and β-endorphin, peptides were extracted from plasma using an extraction kit (Phoenix Pharmaceuticals, Burlingame, CA, USA), following both the manufacturer's instructions and protocols from a previous study^13)^. In brief, 500 μl of plasma was acidified by adding an equal volume of Buffer A (RK-BA-1) and then applied to an SEP-column containing 200 mg of C18 (RKSEPCOL-1). Peptides were eluted with Buffer-B (RK-BB-1), dried using vacuum centrifugation, and stored at −80℃ for later analyses.

### ELISA

β-endorphin and dynorphin A levels were quantified by competitive ELISA using commercial fluorescent EIA kits (Phoenix Pharmaceuticals), following the manufacturer's guidelines. Briefly, the immunoplates in these kits are precoated by secondary antibodies, which bind to the Fc fragment of primary antibodies, the Fab fragment of which is competitively bound by both biotinylated peptides and targeted peptides in samples. Biotinylated peptides interact with streptavidin-horseradish peroxidase (HRP), which catalyzes the substrate solution. Color intensity is directly proportional to the amount of the biotinylated peptide- streptavidin HRP complex, but inversely proportional to the amount of peptides in the standard and samples. Similarly, LCN-2, MMP-9, and MMP- 9/LCN-2 complex levels were measured by sandwich ELISA using fluorescent ELISA Kits (R&D Systems, Minneapolis, MN, USA), in accordance with the manufacturer's instructions. Briefly, the immunoplates in these kits are precoated with a monoclonal antibody for human MMP-9 or the MMP-9/LCN2 complex. MMP-9 or the MMP9/LCN2 complex in the standard and samples bind to the immobilized antibody. After the removal of unbound substances by washing, an enzyme-linked polyclonal antibody specific for human MMP-9 or the MMP-9/LCN2 complex is added to the wells. After the removal of unbound antibody-enzyme reagent by washing, a substrate solution is added to the wells and color develops in proportion to the amount of MMP-9 or the MMP-9/LCN2 complex present.

### Statistical analysis

Statistical analyses were conducted using GraphPad Prism 9 (GraphPad Software, La Jolla, CA, USA). The correlation coefficient (r) was calculated using Pearson's correlation test, with r values classified as weak (0.2 to 0.49), moderate (> 0.4 to 0.7), and strong (> 0.7). The significance of differences was set at *p* < 0.05 in all analyses.

## Results

### Evaluation of plasma dynorphin A and β-endorphin in pruritic skin diseases

**Figure 1:**
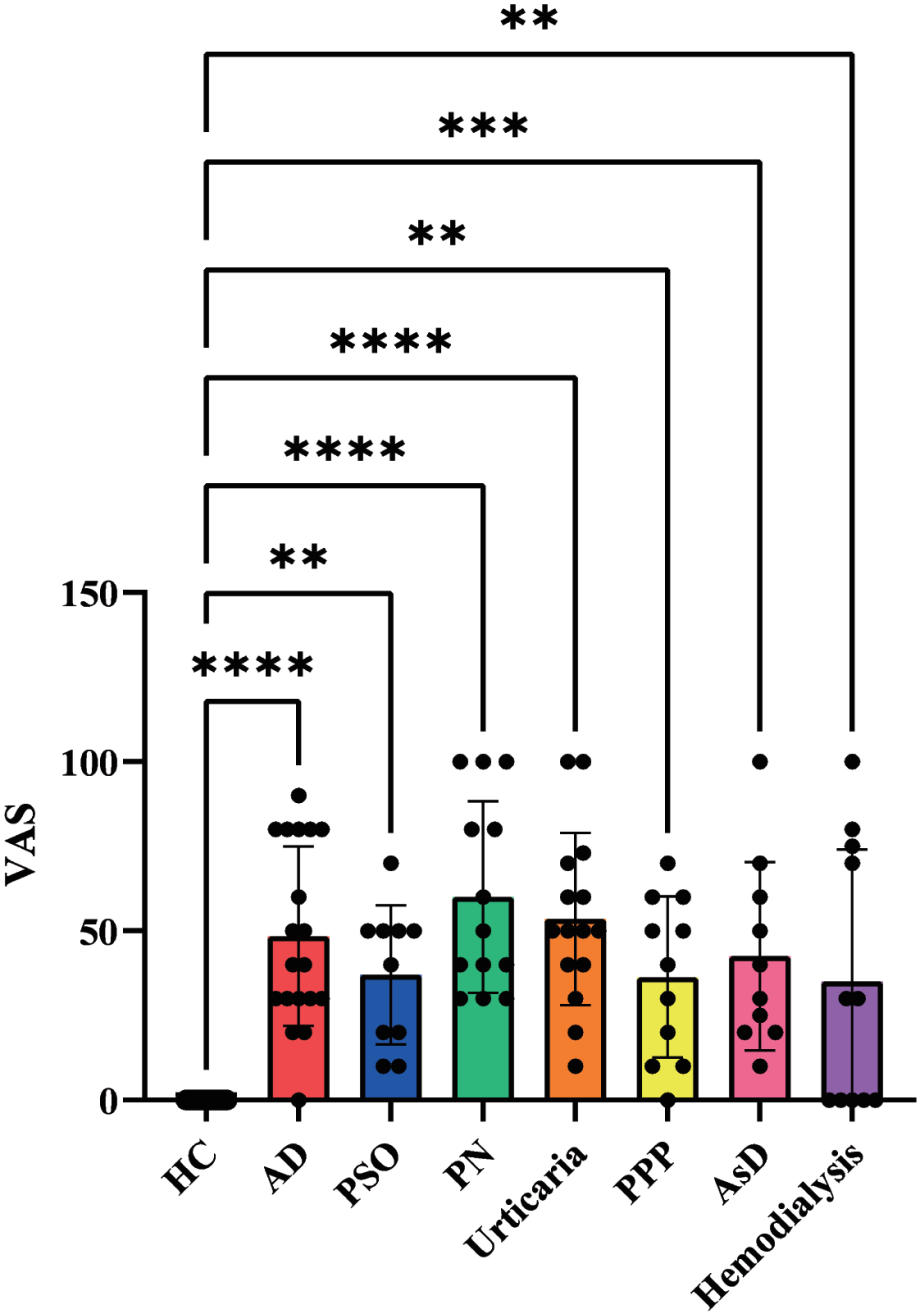
Comparison of VAS scores in healthy controls and patients with pruritic skin diseases VAS scores of 21 healthy controls, 19 patients with atopic dermatitis (AD), 10 with psoriasis vulgaris (PSO), 13 with prurigo nodularis (PN), 15 with urticaria, 11 with palmoplantar pustulosis (PPP), 10 with asteatotic dermatitis (AsD), and 11 receiving hemodialysis were assessed, and the mean scores of each group were compared. **p < 0.01, ***p < 0.001, ****p < 0.0001.

We initially assessed the intensity of itch in patients with pruritic skin conditions, including AD, PSO, PN, urticaria, PPP, and AsD, using VAS. Since opioids play a role in itch modulation in patients receiving hemodialysis^8)^, we also analyzed plasma opioid levels in this group as a positive control, along with VAS scores for itch. Mean VAS scores revealed that the PN group had the highest itch intensity, followed by urticaria, AD, AsD, PSO, PPP, and hemodialysis groups. All disease groups exhibited significantly higher VAS scores than healthy controls (Figure 1).

**Figure 2:**
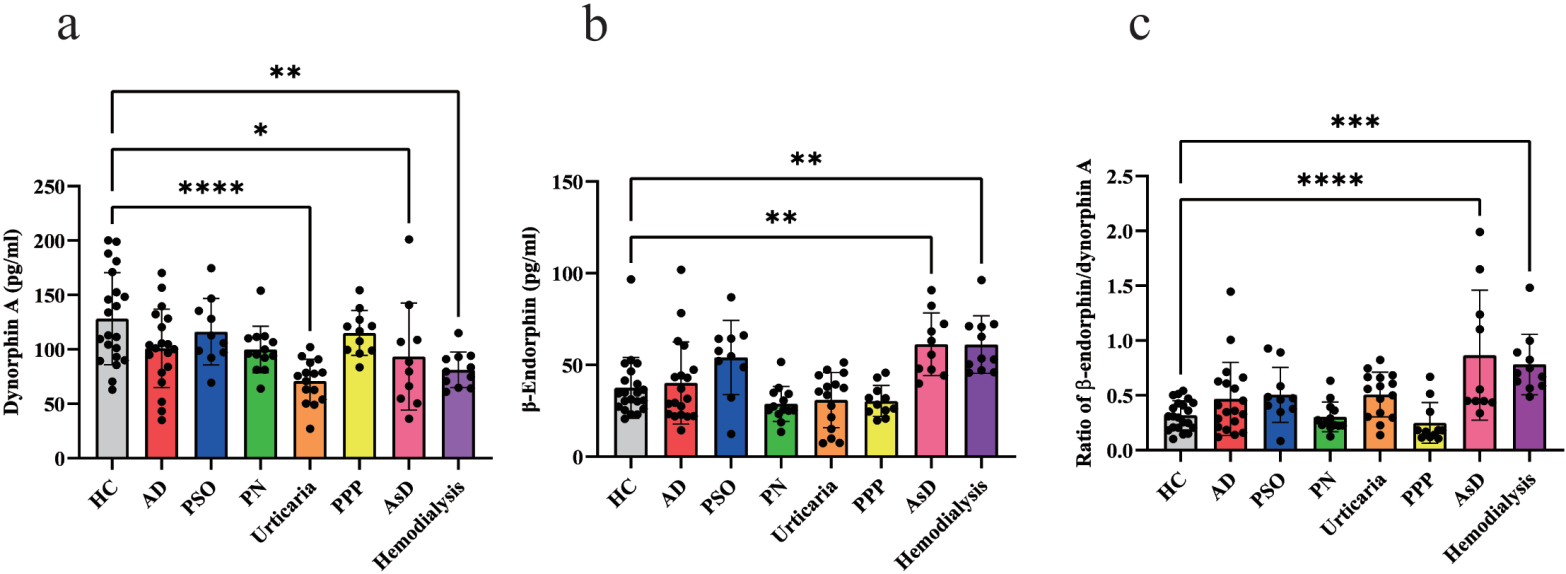
Comparison of plasma dynorphin A levels, β-endorphin levels, and the β-endorphin/dynorphin A ratio in healthy controls and patients with pruritic skin diseases Dynorphin A (a) and β-endorphin (b) levels and the β-endorphin/dynorphin A ratio (c) were measured in the serum of patients with various types of pruritic skin diseases and the mean value of each group was compared with that of healthy controls. *p < 0.05, **p < 0.01, ***p < 0.001, ****p < 0.0001.

We then examined plasma opioid levels in patients with pruritic skin diseases using ELISA. Plasma dynorphin A levels were significantly lower in patients with urticaria and AsD and in those receiving hemodialysis than in healthy controls (Figure 2a). Conversely, plasma β-endorphin levels were significantly higher in patients with AsD and those receiving hemodialysis than in healthy controls (Figure 2b). Furthermore, the β-endorphin/dynorphin A ratio was higher in AsD and hemodialysis patients than in healthy controls (Figure 2c).

### Evaluation of serum LCN-2 in pruritic skin diseases

**Figure 3:**
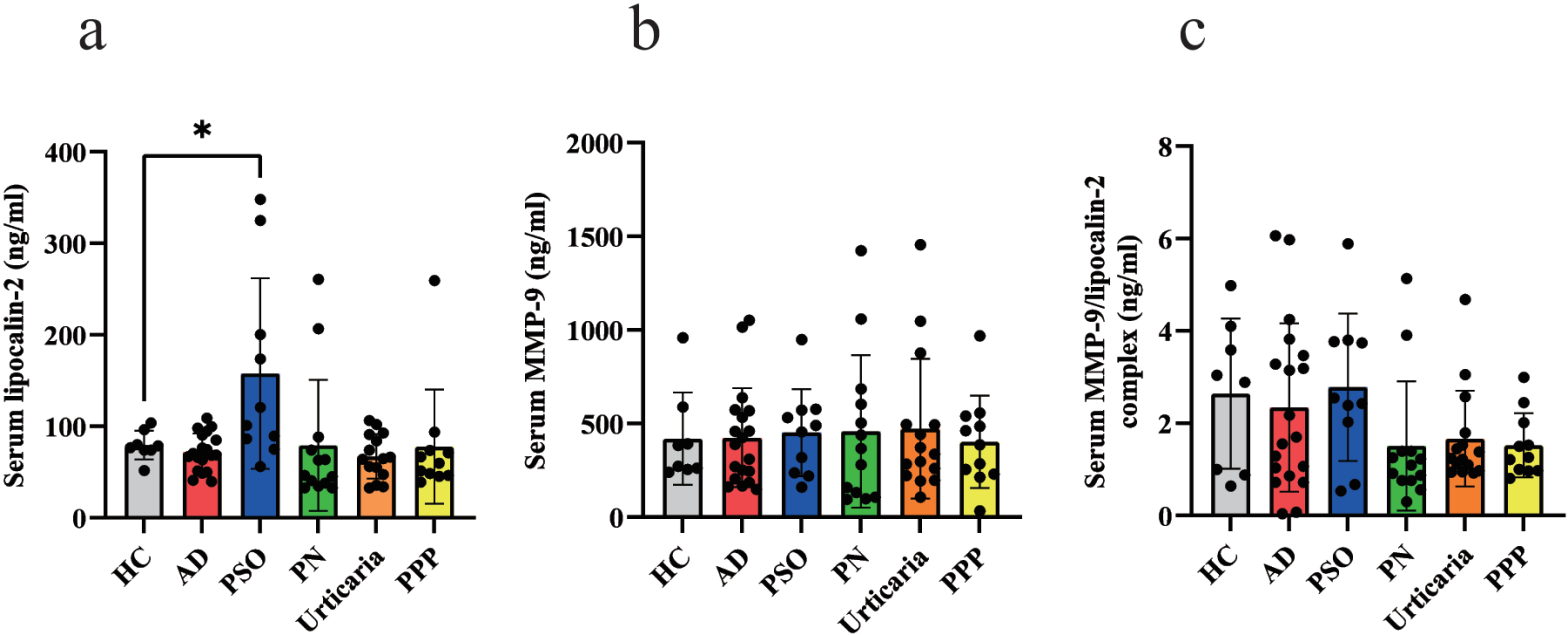
Comparison of serum levels of LCN-2, MMP-9, and the MMP-9/LCN-2 complex in healthy controls and patients with pruritic skin diseases LCN-2 (a), MMP-9 (b), and MMP-9/ LCN-2 complex (c) levels were measured in the serum of eight healthy controls, 19 patients with atopic dermatitis (AD), 10 with psoriasis vulgaris (PSO), 13 with prurigo nodularis (PN), 15 with urticaria, and 11 with palmoplantar pustulosis (PPP), and the mean value of each was compared between groups. *p < 0.05, **p < 0.01.

Since LCN-2 binds to MMP-9 and is present in the bloodstream as the MMP-9/LCN-2 complex^8)^, we assessed serum levels of MMP-9 and the MMP- 9/LCN-2 complex using ELISA. Serum LCN-2 levels were significantly higher in patients with PSO than in healthy controls and those with other pruritic skin conditions, such as AD, PN, urticaria, and PPP (Figure 3a). However, serum MMP-9 and MMP-9/LCN-2 complex levels did not significantly differ among the patient groups or between patients and healthy controls (Figure 3b, 3c).

**Figure 4:**
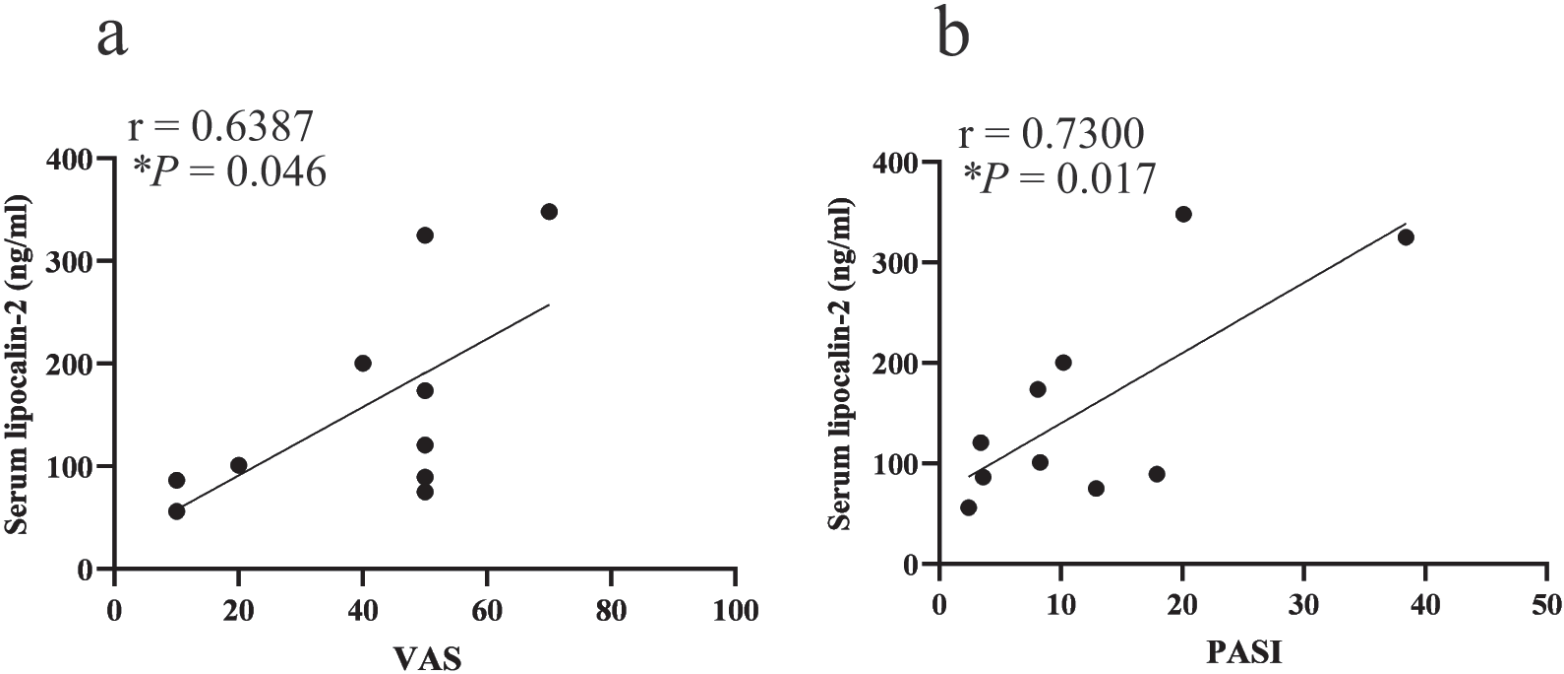
Relationship between serum LCN-2 levels and VAS or PASI scores in patients with psoriasis vulgaris Scatterplots of the relationships between serum LCN-2 levels and VAS scores (a) and serum LCN-2 levels and PASI scores (b) in patients with psoriasis vulgaris. *p < 0.05.

Analyses of the relationships among itch intensity, rash severity, and serum LCN-2 levels in PSO patients revealed that serum LCN-2 levels correlated with VAS scores (Figure 4a) as well as psoriasis area and severity index (PASI) scores (Figure 4b).

## Discussion

In the present study, we examined the potential of plasma opioids and serum LCN-2 levels as objective indicators of itch in patients with pruritic skin diseases. By comparing VAS scores among hemodialysis patients, who served as controls for plasma opioid measurements, and six pruritic skin diseases (Figure 1), we aimed to provide insights into variations in itch intensity across these conditions.

Previous studies on hemodialysis and liver disease cohorts demonstrated that the activation of μ- opioid receptors in the CNS leads to itch, while κ-opioid receptors exert suppressive effects^13-15)^. In the present study, we observed a higher plasma β- endorphin/dynorphin A ratio in AsD and hemodialysis patients than in healthy controls (Figure 2c). However, itch VAS did not correlate with β-endorphin or dynorphin A levels or the β-endorphin/dynorphin A ratio in the pruritic skin diseases examined (data not shown). Blood levels of β- endorphin or dynorphin A may be susceptible to variability and affected by changes in the disease status.

Recent studies indicated that a capsaicin-induced pain stimulation in murine skin activated B5-I neurons (inhibitory interneurons in the dorsal horn of the spinal cord), which subsequently suppressed the itch neurotransmission pathway^16)^. It is hypothesized that B5-I neurons release dynorphin A from nerve endings, inhibiting the ascending itch signaling pathway originating from secondary neurons in the spinal dorsal horn^17)^. Furthermore, epidermal keratinocytes have been shown to express μ- and κ-opioid receptors and their endogenous ligands, and the κ-opioid system was down-regulated in the epidermis of patients with AD and PSO^18, 19)^. A previous study demonstrated that the topical application of a cream containing the μ-opioid receptor antagonist naltrexone effectively reduced itch in AD patients^20)^, indicating the involvement of peripheral opioids in itch modulation. Our research yielded similar findings in mouse models of PSO^21)^. In imiquimod-treated PSO model mice, the topical or intraperitoneal administration of the μ-opioid receptor antagonist naloxone and oral administration of the centrally acting κ-opioid receptor agonist ICI- 199,441 inhibited scratching behavior^21)^. Among the pruritic skin conditions diseases examined, the plasma β-endorphin/dynorphin A ratio was significantly higher in AsD patients than in healthy controls (Figure 2c). Although the specific origins of the opioids in plasma remain unclear, these findings suggest that an activation balance between μ- and κ-opioid receptors partially mediates itch control in AsD patients. Additionally, we observed decreased plasma levels of dynorphin A in urticaria patients, while β-endorphin levels were slightly elevated in PSO patients. Although the β-endorphin/dynorphin A ratio did not significantly differ between urticaria and healthy controls, it was slightly elevated (Figure 2c). This result may reflect an opioid imbalance beginning to emerge in vivo. A recent study reported that the intradermal administration of the μ-opioid receptor agonist endomorphin induced mechanical alloknesis in mice^22)^, further suggesting that β-endorphin or dynorphin A modulates itch, particularly in skin in which opioid levels are changed by disease.

Additionally, we found that serum LCN-2 levels were significantly higher in PSO patients than in healthy controls (Figure 3a). Serum LCN-2 levels correlated with VAS scores for itch and PASI scores in PSO patients (Figure 4), which is consistent with our previous finding showing that serum LCN2 levels correlated with VAS in PSO patients^12)^. However, a relationship was not observed between VAS and serum LCN-2 levels in AD patients, despite our previous finding of a significant increase in serum LCN-2 levels in AD patients^12)^. This discrepancy in AD patients may be attributed to variations in a number of factors, such duration, treatment history, or other clinical characteristics. Among the pruritic skin diseases examined in the present study, only serum LCN-2 levels were significantly higher in PSO patients. Previous studies indicated that astrocytes in the spinal cord dorsal horn of NC/Nga mice with AD were activated and produced LCN-2, contributing to the itch-scratch cycle^23, 24)^. Therefore, while the precise role of LCN- 2 in psoriatic itch has yet to be elucidated, the present results suggest that serum LCN2 levels have potential as a marker for itch and its severity, specifically in PSO patients.

In conclusion, the results obtained herein indicate that the ratio of plasma β-endorphin to dynorphin A is involved in itch control in patients with AsD and those receiving hemodialysis. Furthermore, the absence of a relationship between serum LCN-2 levels and pruritic skin diseases other than PSO suggests that LCN-2 functions as a valuable biomarker for itch and its severity, specifically in PSO patients.

## Funding

No funding was received.

## Author contributions

MK performed all the experiments and writing the manuscript. YK assisted with data analyses and writing the manuscript. EK, CM, and NT assisted with data analyses. TK and YS assisted with the interpretation of experimental data. MT and KT supervised the project, analyzed data, and participated in manuscript writing and review. All the authors have read and approved the final version of the manuscript.

## Conflicts of interest statement

The authors declare that there are no conflicts of interest.
